# Cross‐anatomical evaluation of a deep‐learning auto‐contouring system: qualitative, geometric, and dosimetric validation

**DOI:** 10.1002/acm2.70662

**Published:** 2026-06-15

**Authors:** Sara Endo, Masaki Nakamura, Takeshi Fujisawa, Hidehiro Hojo, Hidenobu Tachibana

**Affiliations:** ^1^ Radiation Safety and Quality Assurance Division National Cancer Center Hospital East Chiba Japan; ^2^ Department of Radiation Oncology National Cancer Center Hospital East Chiba Japan

**Keywords:** deep learning‐based auto‐contouring, dosimetric impact, geometric accuracy, organs at risk (OARs), radiotherapy planning

## Abstract

**Background:**

Accurate and consistent delineation of target and normal structures is essential for safe and effective radiotherapy. Compared with manual or atlas‐based methods, deep learning‐based auto‐contouring has demonstrated improved efficiency and reduced interobserver variability. However, its performance can vary across anatomical regions and clinical contexts, and comprehensive multi‐site validation remains limited. Existing studies have tended to assess only geometric similarity or a single region, providing insufficient insight into real‐world usability and dosimetric influence. A systematic evaluation that integrates physician‐based quality assessment, quantitative geometric metrics, and plan‐level dosimetry across diverse anatomical sites is needed to support broader clinical implementation of deep learning‐based auto‐contouring for differentiation between target and normal structures.

**Purpose:**

To perform the first practice‐oriented, cross‐anatomical evaluation of the SYNAPSE Radiotherapy system (v1.5) and to assess its qualitative usability, geometric agreement (Dice similarity coefficient [DSC], mean distance to agreement [MDA], and maximum Hausdorff distance [HD]), and plan‐level dosimetric impact across six anatomical sites.

**Methods:**

We retrospectively analyzed data for 120 patients across six sites, namely, the head, head and neck, thorax, abdomen, male pelvis, and female pelvis. Thirty organs at risk were auto‐segmented using the SYNAPSE Radiotherapy platform, and three board‐certified radiation oncologists graded the clinical usability of the platform on a 4‐point scale. Geometric agreement was quantified using the DSC, MDA, and maximum HD. Plan‐level dosimetric comparisons were performed for representative targets. Institutional review board approval was obtained (number 2017‐440).

**Results:**

Overall, 76.1% of auto‐contours were qualitatively judged to be clinically usable (score ≥3). Geometric thresholds were achieved for most structures (DSC > 0.8 in 54.8%, MDA < 2.5 mm in 71.5%, and maximum HD < 12.5 mm in 50%), with only 8/29 being major outliers. Auto‐contours tended to be larger than manual contours in 72% of cases. Dosimetrically, 88.1% of organs showed dose‐volume differences within ±5%, and dose constraint achievement rates were comparable between auto‐contours and manual contours (93.9% vs. 95.%). Median doses to planning target volumes (e.g., prostate and iliac nodes) were also consistent, indicating minimal clinical impact.

**Conclusions:**

The SYNAPSE Radiotherapy system showed high overall clinical usability with organ‐dependent variability. Our findings expand the platform‐specific evidence for the SYNAPSE Radiotherapy system by linking geometry to dosimetric impact and supporting clinical deployment with an organ‐specific quality assurance policy, while highlighting contexts in which targeted expert review remains necessary for safe clinical translation.

## 1 INTRODUCTION

Accurate delineation of a target tumor and adjacent normal tissues, known as organs at risk (OARs), is important in radiotherapy planning for achievement of an optimal dose distribution. Precise identification of OARs and avoidance of unnecessary radiation exposure are essential for maximizing tumor control while minimizing the risk of treatment‐related adverse events. Contouring is a critical step in radiotherapy planning, and its accuracy and consistency directly influence the overall quality of treatment.

Manual contouring, however, is time‐consuming and subject to interobserver variability.^1^ Recent advances in deep learning have led to the development of automated contouring technologies that may overcome these limitations. Initial studies focused on automatic delineation of normal organs and demonstrated that deep learning‐based auto‐contouring provides results that are more consistent with physician‐drawn contours than traditional atlas‐based methods, which rely on deformable registration of computed tomography (CT) images.^2^, ^3^, ^4^ These findings have driven the clinical adoption of commercial auto‐contouring tools that incorporate deep learning algorithms. Such tools are expected to streamline clinical workflow, reduce the manual contouring workload, and maintain consistent high‐quality treatment planning across institutions.

Previous studies have evaluated the accuracy of such tools primarily within specific anatomical regions, such as the head and neck or the pelvis, using qualitative, quantitative, and dosimetric metrics.^5^, ^6^, ^7^, ^8^ These metrics include quantitative indices such as the Dice similarity coefficient (DSC) and Hausdorff distance (HD),^4^, ^6^ visual assessments by physicians,^5^ and dosimetric comparisons based on dose distribution.^7^ Collectively, these evaluations suggest that auto‐contouring can improve workflow efficiency and reduce interobserver variability for many normal organs without substantial deviation from physician‐drawn contours.

Nevertheless, discrepancies between automated and physician‐drawn contours can still occur, and consistent accuracy cannot be assumed across all anatomical sites or clinical scenarios. Continued evaluation of the accuracy and reliability of automated contouring, tailored to specific anatomical regions and pathological conditions, is essential to facilitate its broader clinical adoption.^9^

The SYNAPSE Radiotherapy platform (v1.5, Fujifilm Corporation, Tokyo, Japan) is a deep learning‐based radiotherapy planning support system that automatically generates contours for normal organs across a broad anatomical range from the head to the pelvis. However, despite its increasing use in clinical practice, the accuracy of the contours generated by this system and its potential impact on dose distribution have not been systematically evaluated.

Previous investigations of automated contouring have tended to focus on a single anatomical region or on geometric agreement alone,^2^, ^6^ with only a minority including a combination of comprehensive physician‐based qualitative assessment with plan‐level dosimetry.^4^ Among the multi‐region analyses, we identified only one study spanning the head, thorax, abdomen, and pelvis^8^; however, its dosimetric evaluation was restricted to dose‐volume indices, for example, the mean dose (D_mean_) and the highest dose delivered to any 2 cc (D_2cc_), which may not fully capture the clinical impact across the various organ classes. Therefore, there is a need for an evaluation framework that couples physician usability ratings with orthogonal geometric metrics (e.g., the DSC, mean distance to agreement [MDA], and HD) and clinically interpretable dosimetric endpoints linked to organ‐specific acceptability thresholds.

In this study, we performed a practice‐oriented, cross‐anatomical evaluation of the SYNAPSE Radiotherapy system. This research constitutes the first platform‐specific assessment of the qualitative, geometric, and dosimetric performance of this system across six anatomical regions.

## 2 METHODS

### 2.1 Patient data

This retrospective study included 120 patients who underwent radiotherapy at our institution between June 2022 and March 2024, comprising 20 patients for each of six anatomical regions (head, head and neck, thorax, abdomen, male pelvis, and female pelvis). Institutional review board approval was obtained (approval number 2017‐440). The requirement for informed consent was waived owing to the retrospective nature of the study.

### 2.2 Image acquisition

CT simulation was performed in all cases using an Aquilion ONE PRISM 320‐slice CT scanner (Canon Medical Systems Inc., Otawara, Tochigi, Japan). Patients were positioned head‐first supine, and images were acquired with a slice thickness of either 1 or 2 mm. The imaging data were then transferred to the respective treatment planning systems.

### 2.3 Contouring and treatment planning

Treatment was planned using the RayStation system (v2023B, RaySearch Laboratories, Stockholm, Sweden) and the Eclipse system (v16.00.00, Varian Medical Systems, Palo Alto, CA, USA). For each patient, anatomical structures were manually delineated on planning CT scans using Eclipse and MIM Maestro (v7.4, MIM Software Inc., Cleveland, OH, USA) in accordance with institutional clinical protocols. Although contouring was performed by personnel with varying levels of experience, all structures were reviewed and approved by senior radiation oncologists, each with over 10 years of experience. The required anatomical structures differed from patient to patient because of variability in tumor sites.

### 2.4 Automatic contouring workflow

Thirty automatic organ contours were generated using the SYNAPSE Radiotherapy (v1.5) platform, (Table 1). This system incorporates both deep learning‐based and conventional machine learning‐based approaches. According to information provided by the vendor, structures developed using deep learning are based on U‐Net‐type architectures. In contrast, several structures, including the body contour (threshold‐based), vertebrae, brain, bronchial tree, and pulmonary vessels, are generated using conventional (non‐deep learning) methods. Following image acquisition, the CT data were transferred to SYNAPSE Radiotherapy, where organ contours were automatically generated for each anatomical region. The automatically generated contours were used without any range specification or manual modification. Institutional contouring definitions, such as region‐specific start and end slice conventions or margin‐based extensions, were intentionally not applied, so that the intrinsic unadjusted performance of the algorithm could be evaluated. This approach was chosen to evaluate the intrinsic performance of the software.

**TABLE 1:** Distribution of cases and structures across qualitative, quantitative, and dosimetric evaluations.

Structure name	Region	Number of data			Structure name	Region	Number of data		
		Qualitative	Quantitative	Dosimetric			Qualitative	Quantitative	Dosimetric
Brainstem	Head, Head, and Neck	20	35	53	Kidney_L	Abdomen	20	18	3
Brain	Head, Head, and Neck	20	31	—	Kidney_R	Abdomen	20	18	3
Chiasm	Head, Head, and Neck	20	19	7	Stomach	Abdomen	20	14	24
Eye_L	Head, Head, and Neck	20	28	8	Ascending Aorta	Abdomen	20		
Eye_R	Head, Head, and Neck	20	27	8	Liver	Abdomen	20	18	30
Optic nerve_L	Head, Head, and Neck	20	26	6	Pancreas	Abdomen	20		
Optic nerve_R	Head, Head, and Neck	20	24	6	Duodenum	Abdomen	20	13	19
Lens_L	Head, Head, and Neck	20	10	5	Bladder	Male pelvis, female pelvis	40	40	116
Lens_R	Head, Head, and Neck	20	10	5	Femoral Head_L	Male pelvis, female pelvis	40	20	7
Spinal cord	Head, Head, and Neck	80	69	89	Femoral Head_R	Male pelvis, female pelvis	40	20	7
	Thorax, Abdomen								
Mandible	Head and Neck	20	11	10	LN_Iliac	Male pelvis, female pelvis	40	25	25
Parotid_L	Head and Neck	20	12	11	Rectum	Male pelvis, female pelvis	40	40	197
Parotid_R	Head and Neck	20	11	10	Prostate	Male pelvis	20	19	19
Esophagus	Thorax	20	26	47	Seminal Vesicle	Male pelvis	20	8	—
Heart	Thorax	20	28	63	Total		760	640	880
Lung	Thorax	20	20	102					

Abbreviations: L, left; LN, lymph node; R, right.

### 2.5 Qualitative assessment

Three board‐certified radiation oncologists independently evaluated the automatically generated contours using the following four‐point scoring system: 4, only minimal editing required for clinical use; 3, minor editing involving three or fewer slices; 2, editing required for four or more slices; 1, complete redrawing of the region required.^5^ The scoring criteria were based on the number of slices requiring manual correction and overall clinical usability. All evaluations were performed in a blinded manner.

### 2.6 Quantitative evaluation

Agreement between the automated and manual contours was assessed using the DSC, MDA, and HD. The DSC is a metric that measures the spatial overlap between two structures and ranges from 0 (no overlap) to 1 (perfect agreement).^10^ The MDA represents the average distance between two contour surfaces, with lower values indicating better agreement.^11^ HD represents the maximum distance between surface points on the contours, with lower values indicating better agreement.^9^, ^12^

For each case, 28 structure pairs were analyzed (Table 1). The Ascending Aorta was excluded because it was not involved in the treatment plan, and the Pancreas was omitted in view of the absence of manual contouring and dose constraints. The DSC, MDA, and HD were calculated for the remaining 28 structures, and the results were grouped by anatomical region (head, head and neck, thorax, abdomen, and pelvis) to evaluate regional trends.

### 2.7 Dosimetric evaluation

Dose‐volume histograms (DVHs) were calculated using both manual and automatic contours, and dosimetric indices were compared for 27 organs in accordance with institutional protocols. The indices evaluated included the D_mean_, maximum dose (D_max_), and, where appropriate, volume‐based metrics such as V20Gy.

The Ascending aorta and Pancreas were excluded for the reasons mentioned earlier (Table 1). The Seminal vesicles were also excluded from the dosimetric analysis because they were not subject to any dose constraints. For each case, the original planning CT and physician‐approved treatment plan were retained, with only the contours of the evaluated organs replaced by their automatically generated versions. The DVHs were subsequently recalculated, and the resulting dosimetric parameters were compared to evaluate the impact of automatic contouring.

## 3 RESULTS

Thirty organ contours were automatically generated using the SYNAPSE Radiotherapy system and evaluated from the following three perspectives: qualitative scoring by physicians; quantitative evaluation (DSC, MDA, and HD); and assessment of the impact on dose distribution (DVH analysis).

### 3.1 Qualitative assessment by physicians

**FIGURE 1:**
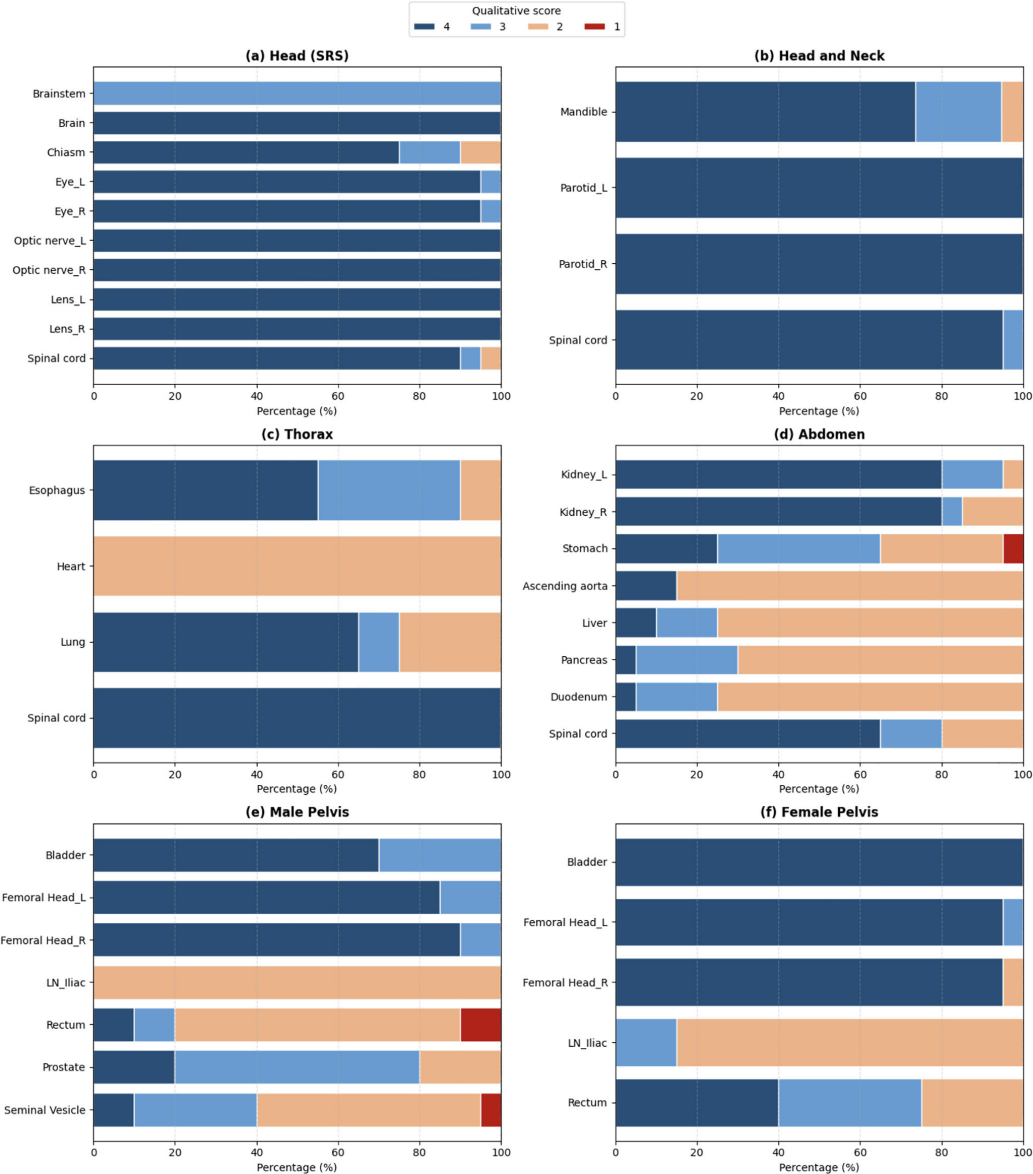
Results of qualitative scoring for auto‐contouring evaluation. (a) Head (SRS), (b) Head and neck, (c) Thorax, (d) Abdomen, (e) Male pelvis, and (f) Female pelvis. The distribution of qualitative scores (1–4) is shown for each organ. SRS, stereotactic radiosurgery.

Three radiation oncologists qualitatively assessed the 30 automatically generated organ contours using the four‐point scoring system. The results are shown in Figure 1. Overall, 76.1% of the contours received a score of 3 or 4, indicating that they were either clinically acceptable without modification or required only minor adjustments.

In the brain and the head and neck regions, all organs achieved an average score of ≥3, reflecting generally high accuracy in auto‐contouring for these anatomical sites. Specifically, the Brain, Lens_L/R, Optic Nerve_L/R, and Parotid_L/R consistently received a score of 4, suggesting that they were clinically acceptable without needing revision. In the thoracic region, the Esophagus, Lungs, and Spinal cord had average scores of ≥3, indicating strong geometric and anatomical consistency with manual contours.

In contrast, the Heart consistently received a score of 2 across all cases, highlighting the need for further manual refinement. In the abdominal region, the Kidney_L/R and Spinal cord received average scores of >3, indicating their clinical value with minimal edits. Conversely, more anatomically complex abdominal structures, such as the Stomach, Ascending Aorta, Liver, Pancreas, and Duodenum, had lower scores (ranging from 2.3 to 2.9), suggesting that additional manual correction was often necessary. Within the male and female pelvic regions, structures with ambiguous anatomical boundaries, such as the Rectum (scores 2.2–3.2) and Lymph Nodes (scores 2.0–2.2), tended to receive lower evaluations.

However, the Bladder, Femoral Head_L/R, and Prostate were generally rated as acceptable with only minor modifications. Notably, the Spinal cord was included across multiple preset regions. Its average qualitative scores showed minimal variation (Brain, 3.9; Head and Neck, 4.0; Lung, 4.0; Liver/Pancreas, 3.5).

### 3.2 Quantitative evaluation

**FIGURE 2:**
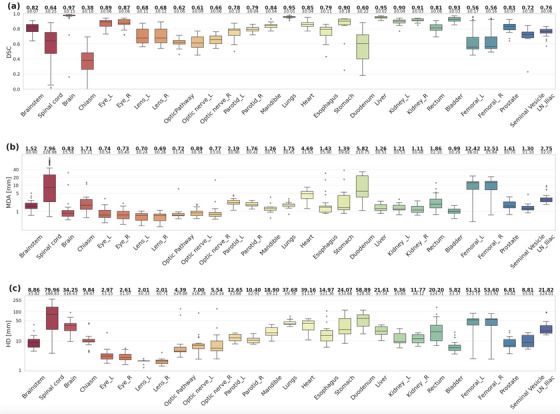
Quantitative evaluation of auto‐contouring accuracy across organs. (a) Dice similarity coefficient (DSC). (b) Mean distance to agreement (MDA). (c) Maximum Hausdorff distance (HD).

Twenty‐nine automatically generated organ contours were quantitatively evaluated using the DSC, MDA, and HD (Figure 2). Structures demonstrating high spatial agreement as measured by the DSC included the Brain and Eye_L/R in the brain region, the Lungs in the thoracic region, the Liver and Kidney_R in the abdominal region, and the Bladder in both the male and female pelvic regions. These structures achieved average DSC values ranging from 0.9 to 1.0. In contrast, lower DSC values (0.3–0.6) were observed for the Spinal cord and Chiasm in the brain region, the Chiasm in the head and neck region, the Duodenum in the abdominal region, and the Femoral Head_L/R in the male pelvic region. The proportion of structures with a DSC of <0.7 was 29.1%. These low values were more frequently observed in small‐volume structures, particularly in the head and neck region. Furthermore, 7.8% of structures showed a DSC of <0.5, with the majority of these cases involving the Spinal cord and Chiasm. These findings indicate that a non‐negligible proportion of cases showed segmentation limitations that may be clinically relevant depending on the anatomical context.

For MDA, high overall agreement was observed in the brain and the head and neck regions, particularly for the Brainstem, Eye_L/R, and Mandible, with average MDA values between 0.6 and 2.4 mm. However, larger discrepancies were identified in the Spinal cord (excluding the abdominal preset), Duodenum, and Femoral Head_L/R, where average MDA values exceeded 10 mm, indicating substantial deviations from the manual contours.

Most organs in the brain and the head and neck regions had maximum HD values in the range of 2.0–11.1 mm, suggesting the absence of major outliers. Similarly, relatively large structures such as the Kidney_L/R in the abdomen and the Bladder, Prostate, and Seminal vesicles in the pelvic region had HD values between 6.1 and 12.5 mm, indicating high consistency between automatic and manual contours. However, a small number of outliers were observed, particularly in the brain and the head and neck regions, where the HD exceeded 100 mm in some cases. Furthermore, the large size of the abdominal organs often resulted in higher HD values even when the spatial deviation was minimal. MDA values of >5 mm were observed in 16.5% of structures, and HD values of >50 mm in 15.3%. These were more frequently observed in elongated structures, such as the Spinal cord. Overall, 54.8% of the auto‐segmented structures achieved a DSC of >0.8, 71.5% achieved an MDA of <2.5 mm, and 50% achieved an HD of <12.5 mm, indicating that the majority of structures reached an acceptable level of agreement for clinical use. There were a few outliers with abnormal metric values, where parts of the target structure were completely missing or where adjacent non‐target structures were mistakenly included. Segmentation failures were observed in structures such as the Optic nerve (*n* = 3), Eye (*n* = 2), Mandible (*n* = 2), and Stomach (*n* = 2).

Analysis of the consistency between qualitative scores and quantitative indices revealed that among the 433 structures with a qualitative score of ≥3 and for which both manual and automatic contours were available, a DSC of >0.8 was achieved in 244 cases (56.4%). Furthermore, the automatic contours were larger than the manual contours in 72% of the structures. The greatest volume increase was observed in the Chiasm (+2.68). Despite favorable qualitative scores (3 or 4) assigned by physicians, a subset of cases were found to have poor quantitative metrics (DSC, HD, and MDA) across multiple organs, indicating that contours considered visually acceptable could still show substantial geometric discrepancies. These findings suggest that minor differences deemed to be clinically acceptable on visual assessment may be sensitively detected by quantitative evaluation.

### 3.3 Evaluation of dosimetric impact

Dosimetric evaluation of the 29 automatically segmented organs was performed using DVH analysis. Institutional protocol‐defined dose constraints were applied to both manually and automatically generated contours. The differences in dose and volume metrics between the two methods were assessed, along with whether each structure met or failed to meet the specified dose constraints (Tables S1–S6).

A dose and volume difference within ±5% was found for 88.1% of the evaluated structures, indicating strong dosimetric consistency between the auto‐segmented and manual contours for the majority of organs. The overall constraint achievement rate was 9 3.9% for auto‐contouring and 95.1% for manual contouring.

In total, 24.6% of dose constraint violations occurred only in the auto‐segmented contours, 1.7% occurred only in the manual contours, and 73.7% were common to both contours. Among the 887 dose comparisons performed, the dose based on auto‐contours was underestimated in 303 cases (34.2%), overestimated in 474 (53.4%), and unchanged in 112 (12.6%). According to the dose constraint graphs, the dose was underestimated using auto‐contours in 63.6% of cases and overestimated in 32.7%. In 3.6% of cases, there was no change in dose between manual contours and auto‐contours. The overall correlation between manual and automatic dose estimations was 78.2%, indicating strong agreement.

Representative dose‐volume relationships for each anatomical region are shown in Figures S1–S6 and demonstrate high concordance in the Brain and Abdomen, with greater variability for the Chiasm (head and neck), Heart (thorax), and Rectum (male pelvis).

In the pelvic region, additional dose comparisons were performed for planning target volumes generated by expanding the prostate and lymph node structures with institutional margins, specifically, the lymph nodes in female patients and the prostate and lymph nodes in male patients (evaluated by the median dose [D50%]).

## 4 DISCUSSION

This study evaluated the accuracy and clinical applicability of contours generated by SYNAPSE Radiotherapy, a deep learning‐based auto‐contouring system, in 120 cases. The evaluation encompassed three aspects: qualitative evaluation, quantitative assessment (DSC, MDA, HD), and dosimetric evaluation. To our knowledge, as of September 3, 2025, this is the first peer‐reviewed cross‐anatomical evaluation of the SYNAPSE Radiotherapy (v1.5) platform that links geometric agreement with plan‐level dosimetric differences. Nevertheless, the conclusions should be interpreted primarily as implementation guidance for clinical deployment, rather than as a generalized validation of the algorithm. The automated contours for many organs were consistently accurate. However, our results also highlight a need for modifications arising from differences in organ morphology, spatial relationships, and contouring definitions. In the brain and the head and neck regions, minor corrections (≤3 slices) were frequently required for the brainstem because of small contour omissions at the superior edge and partial overlap with the Spinal cord at the inferior edge. Small‐volume structures, such as the Chiasm and Optic pathways, had lower DSC values, but the MDA and HD remained within clinically acceptable limits. Overall, these inconsistencies were attributed primarily to differences in the definition of the delineation range rather than failure of the algorithm. In the Spinal cord, numerous outliers exceeding 150 mm in HD occurred as a result of discrepancies between the full length delineated by the automatic contours and the clinically required range delineated manually.

Even minor positional shifts can markedly reduce the DSC for organs with a small volume. However, if the MDA and HD, which represent spatial agreement, remain within acceptable ranges, the clinical impact may be limited depending on the characteristics of the organ and its spatial relationships. However, owing to the steep dose gradient around certain organs and the proximity of critical structures, even slight misalignments may have substantial dosimetric consequences. Clinically, the impact of geometric errors varies depending on the characteristics of the OAR. In small or serial organs, such as the chiasm, optic nerve, spinal cord, and duodenum, even minor contouring errors can lead to substantial dosimetric differences. In relatively large organs, such as the esophagus and rectum, the influence of hotspots should be considered. In contrast, for large parallel organs, such as the liver and lungs, the impact of geometric errors is likely to be relatively limited, considering that these structures are typically evaluated based on volumetric averages. Therefore, it is essential not to rely solely on quantitative metrics but to integrate dose evaluation, with final confirmation ultimately required by the physician's review.

In the dose evaluation, most organs demonstrated extremely high correlations and regression slopes close to 1, indicating strong geometric and dosimetric consistency between dose estimates based on automatic and manual contours. However, for the Brainstem $D_{1cm^{3}}$, the regression slope was less than 1 [f(x) = 0.55x + 0.90], suggesting a tendency for automatic contours to underestimate dose. This implies that the Brainstem may not have been fully encompassed by the automatic segmentation, raising concerns about potential underestimation of the dose to be delivered. Across 887 evaluations, 303 (34.2%) showed underestimation and 474 (53.4%) showed overestimation, with no difference in 112 (12.6%). Many underestimations were attributable to contour omissions at the superior and inferior margins or to insufficient volume delineation. The dose‐constraint achievement rates in this study (93.9% for auto‐contours and 95.1% for manual contours) were similar to those reported by Douglas et al.,^7^ thereby confirming the reproducibility and clinical applicability of our findings.

In the qualitative assessment of the thorax region, heart contours received lower scores because of differences in definition between the SYNAPSE Radiotherapy platform and manual contouring at our institution. These inconsistencies stemmed primarily from variations in setting the superior and inferior contour limits and differences in recognizing boundaries with adjacent tissues. In the quantitative assessment, variability in the DSC was observed for the Spinal cord. Similar to the findings in the head and neck region, some cases showed an increased HD as a consequence of differences in the extent of contour delineation. In the dose evaluation, automated contouring resulted in over‐delineation of organs in certain cases, leading to larger volume assessments and consequently higher calculated doses. Comparable trends were observed in the esophagus, a long tubular structure with a steep dose gradient, and in the eart, where we found differences in definition between the SYNAPSE Radiotherapy system and our manual delineation. Corrections are necessary for these organs to ensure the accuracy of dose distribution.

In the qualitative evaluation of the abdominal region, the highest proportion of cases required corrections corresponding to a score of 2, involving four or more slices. Discrepancies at the superior and inferior margins, as well as frequent overlaps with adjacent organs, were common, indicating limitations in the accuracy of automatic contouring. In the quantitative evaluation, DSC values remained at a moderate level; however, considerable variability was noted in MDA and HD across many organs, likely reflecting the relatively large size and complex morphology of abdominal structures. In a study by Ahn et al.,^3^ deep learning‐based auto‐segmentation also yielded favorable DSC values for the Kidney, Liver, and Stomach; however, in the present study, these organs had even higher DSC values, indicating superior performance of the algorithm. In particular, relatively accurate contours were obtained for the Stomach, an organ with substantial intra‐fraction and inter‐fraction variability, demonstrating the robustness of the algorithm. In the dose evaluation, the abdominal organs are often classified as OARs subject to strict dose constraints, and inaccurately defined boundaries can result in overestimation or underestimation of the dose. Dose assessments from automatic and manual contours showed high concordance across many organs and metrics, with clinically reliable accuracy confirmed for the Spinal cord, Duodenum, Kidney, and Liver in particular. However, for the Heart, differences in definition led to systematic overestimation of volume in the automatic contours, resulting in dose overestimation. Although this finding was most pronounced for the Heart, similar inaccuracies in the superior and inferior margins or missing contours could lead to overestimation or underestimation of volume in other abdominal organs. Therefore, careful confirmation remains necessary when applying automatic contouring in clinical practice, regardless of the target organ.

In the pelvic region, all three metrics, namely, DSC, MDA, and HD, showed good agreement for the bladder and prostate. Specifically, the DSC values for the bladder and prostate averaged 0.92 and 0.82, respectively, indicating high accuracy. For organs with distinct and well‐defined shapes, the model appeared to provide relatively stable contour delineation. In contrast, for lymph nodes, variability in volume and ambiguous boundaries led to a wide range of DSC values (0.57–0.83), with less favorable results also observed for the MDA and HD. This reflects the inherent difficulty of defining and recognizing lymph nodes in the automatic contouring model and highlights the fact that human confirmation remains indispensable at this stage.

Comparison of the D_50%_ with the planning target volume showed that dose values derived from automated and manual contours were in good agreement with the planning target volumes for Prostate and LN_Iliac created with our institutional margins. The planning differences remained within quantitatively consistent ranges. These findings indicate that automated contouring has reached a level suitable for practical clinical application.

Overall, automated contours were qualitatively judged to be clinically usable (score ≥3) for 76.1% of structures, with a DSC of >0.8 achieved in 54.8% of cases, an MDA of <2.5 mm in 71.5%, and an HD of <12.5 mm in 50%. Outliers exceeding 100 mm in HD were observed in eight of the 29 structures evaluated, with the Spinal cord reaching up to 150 mm. Among the 433 structures that received a qualitative score of ≥3, 244 (56.4%) had a DSC of >0.8, demonstrating consistency between qualitative assessments and quantitative metrics. These results indicate that, while automated contouring has a high overall success rate, organ‐specific characteristics define areas where particular caution is required.

This study consistently showed that structures with distinct shapes and boundaries (e.g., the bladder, eye, and liver) were more likely to show strong agreement, whereas accuracy tended to decrease for long tubular structures (e.g., the esophagus and spinal cord), small‐volume structures (e.g., the chiasm and optic nerve), and structures with definitions that vary across institutions (e.g., the heart and lymph nodes). Consistent with previous reports,^3^, ^5^, ^7^, ^8^ these findings provide useful evidence to guide target selection and development of operational policy for the clinical implementation of automated contouring. Automatic contours were evaluated without application of range specification or manual adjustment or in alignment with institutional definitions so that the intrinsic performance of the software could be evaluated. This choice of design likely contributed to the reduced accuracy observed for certain organs, particularly those with a long tubular shape, a small volume, or an institution‐dependent definition. Cases were limited for some organs, which may have affected the robustness of statistical comparisons. This shortcoming reflects the distribution of clinical cases and should be considered when interpreting the results. Each anatomical region was evaluated by a designated radiation oncologist, and independent multi‐observer evaluation of the same cases was not performed. Therefore, interobserver variability could not be assessed in this study and remains an important topic for future investigation. Nevertheless, the SYNAPSE Radiotherapy system provides functionality for range specification and customization of definition, and its accuracy is expected to improve when such settings are applied in clinical practice.

Finnegan et al. recently published a multicenter evaluation of artificial intelligence (AI)‐based auto‐contouring using the Ethos system (Varian Medical Systems).^8^ Ethos demonstrated good agreement for distinct structures such as the Bladder, Liver, Lungs, and Femoral head (signal DSC > 0.9, MDA < 2.5 mm). In contrast, significant geometric errors were observed for indistinct, long tubular, or small‐volume organs such as the Duodenum, Optic Nerve, and Spinal cord, which is a trend consistent with the findings for the SYNAPSE Radiotherapy system in the present study. The Ethos system also achieved a signal DSC of >0.8 in more than 70% of structures, which is comparable with the DSC of >0.8 in 54.8% of structures in the present study. Moreover, the Ethos study by Finnegan et al. found maximum dose differences exceeding 30 Gy for the D_2cc_ and D_mean_ in certain structures, highlighting the fact that dose consistency and management of outliers continue to be common challenges. The trend for alignment of accuracy among organs with similar contouring characteristics (e.g., distinct boundaries vs. ambiguous margins) suggests that the distinction between easily delineated and difficult‐to‐delineate structures is a common feature of AI‐based contouring systems.

Based on these comparisons, the SYNAPSE Radiotherapy system can be considered to have performance equivalent to that of other commercially available AI tools. However, as with the Ethos system, careful selection of applicable structures and regular quality assurance remain essential, and these findings should serve as a reference for decisions regarding implementation at specific institutions.

## 5 CONCLUSIONS

Automated contouring using the SYNAPSE Radiotherapy system demonstrated high geometric and dosimetric accuracy across multiple organs, with potential benefits in terms of improving efficiency and reducing inter‐operator variability. However, discrepancies and errors remain for anatomically complex or high‐risk structures, underscoring the ongoing need for expert review. The findings of this study clarify the current effectiveness and limitations of auto‐contouring technology and provide a foundation for its safe clinical implementation. Future studies should explore adaptive and longitudinal applications of this system, including its integration into planning adaptation online and quality assurance workflows.

## AUTHOR CONTRIBUTIONS

Hidenobu Tachibana conceptualized the study, helped interpret the data, and provided expertise in manuscript preparation. Sara Endo developed the study design and analyzed data. Masaki Nakamura, Takeshi Fujisawa, and Hidehiro Hojo evaluated the contours of normal structures. Hidenobu Tachibana and Sara Endo critically revised the manuscript.

## CONFLICT OF INTEREST STATEMENT

All authors declare no conflict of interest.

## ETHICS STATEMENT

This retrospective study was approved by the institutional review board of National Cancer Center Hospital East (2017‐440). The requirement for informed consent was waived due to the retrospective nature of the study.

## Supporting information




**Supporting Information**: 2026‐09190‐sup‐0002‐SI_Figure‐S01.pdf

**Supporting Information**: 2026‐09190‐sup‐0003‐SI_Figure‐S02.pdf

**Supporting Information**: 2026‐09190‐sup‐0004‐SI_Figure‐S03.pdf

**Supporting Information**: 2026‐09190‐sup‐0005‐SI_Figure‐S04.pdf

**Supporting Information**: 2026‐09190‐sup‐0006‐SI_Figure‐S05.pdf

**Supporting Information**: 2026‐09190‐sup‐0007‐SI_Figure‐S06.pdf

**Supporting Information**: 2026‐09190‐sup‐0008‐SI_Table‐S01.pdf

## Data Availability

The data that support the findings of this study are available from the corresponding author upon reasonable request.
