# Supplementary material for: Cross‐anatomical evaluation of a deep‐learning auto‐contouring system: qualitative, geometric, and dosimetric validation
Source: J Appl Clin Med Phys. 2026 Jun 15;27(6):e70662. doi: 10.1002/acm2.70662 (PMC13269653; doi:10.1002/acm2.70662)
Supplement: Supplementary file 1 — Supporting Information: 2026‐09190‐sup‐0002‐SI_Figure‐S01.pdf [file ACM2-27-e70662-s003.pdf]

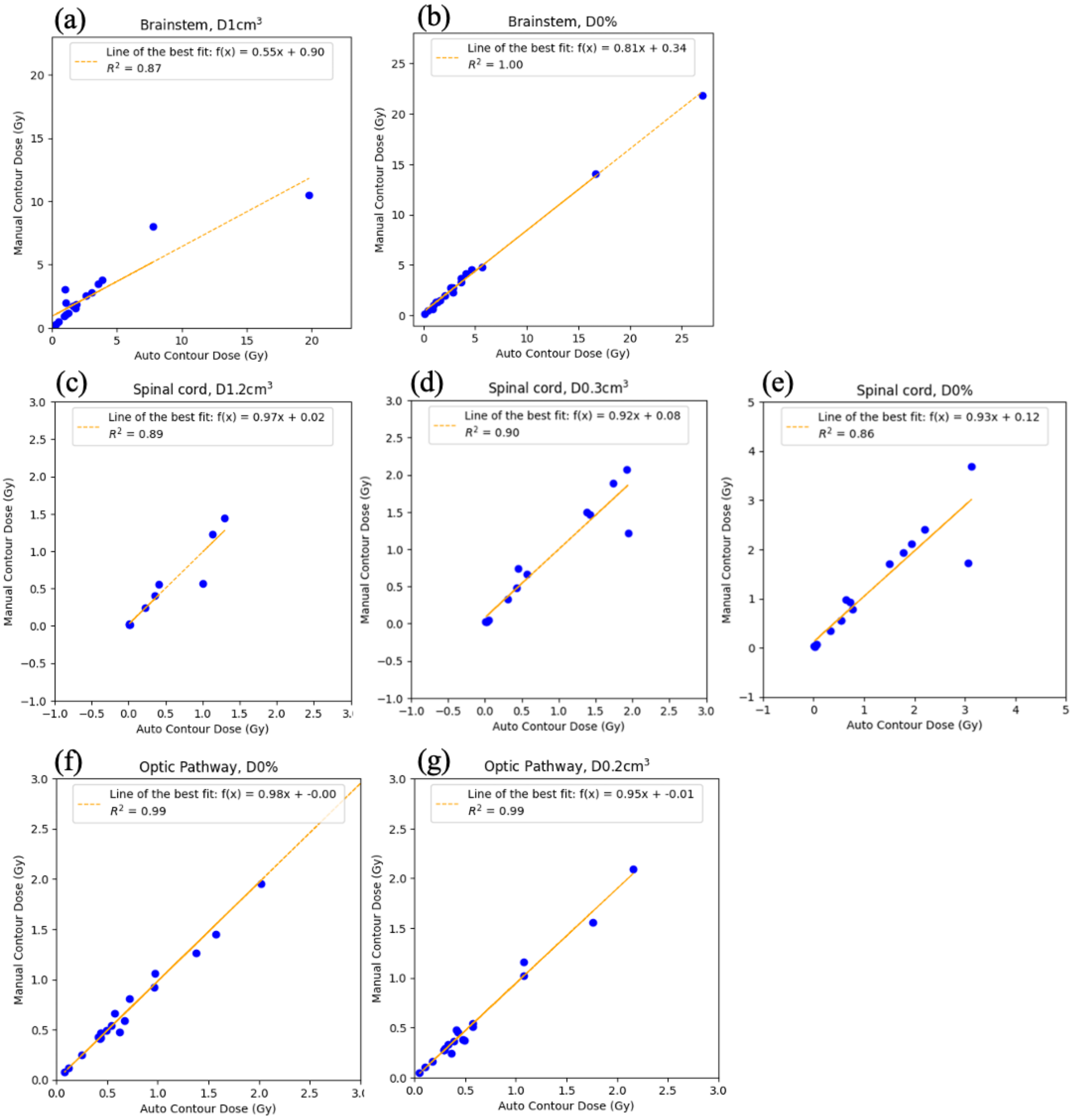

Supplementary Fig. 1 Scatter plots comparing auto-contoured and manually contoured doses for organs when planning stereotactic radiosurgery to the brain. (a) Brainstem ($D1cm^3$), (b) Brainstem ($D_{0\%}$), (c) Spinal cord ($D_{1.2cm^3}$), (d) Spinal cord ($D_{0.3cm^3}$), (e) Spinal cord ($D_{0\%}$), (f) Optic pathway ($D_{0\%}$), and (g) Optic pathway ($D_{0.2cm^3}$). Each panel shows the line of best fit with its regression equation and coefficient of determination (R^2).
