# Supplementary material for: Cross‐anatomical evaluation of a deep‐learning auto‐contouring system: qualitative, geometric, and dosimetric validation
Source: J Appl Clin Med Phys. 2026 Jun 15;27(6):e70662. doi: 10.1002/acm2.70662 (PMC13269653; doi:10.1002/acm2.70662)
Supplement: Supplementary file 2 — Supporting Information: 2026‐09190‐sup‐0003‐SI_Figure‐S02.pdf [file ACM2-27-e70662-s006.pdf]

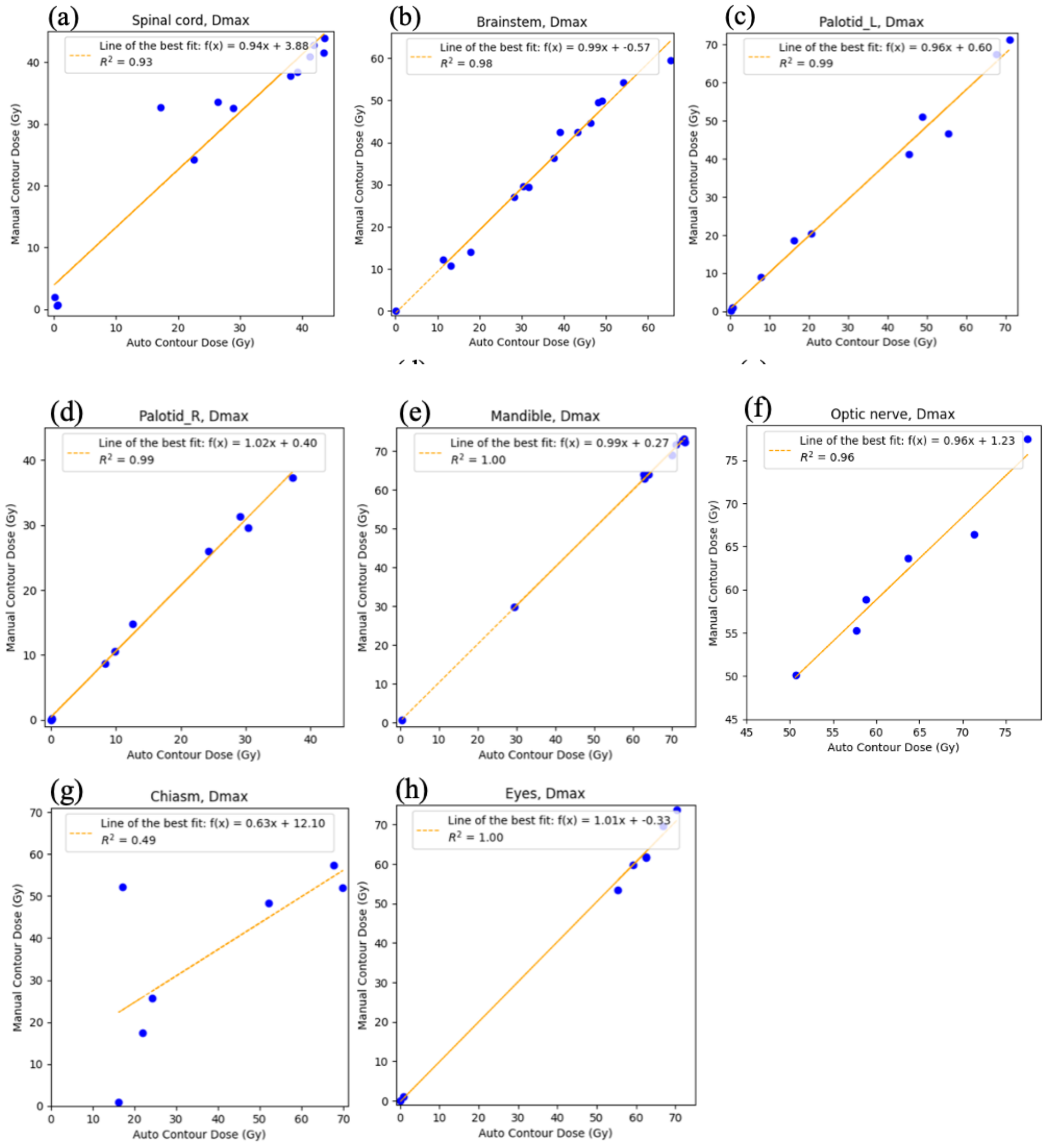

Supplementary Fig.2 Scatter plots comparing auto-contoured and manually contoured doses for organs at risk when planning radiotherapy in the head and neck region. (a) Spinal cord, (b) Brainstem, (c) Left parotid, (d) Right parotid, (e) Mandible, (f) Optic nerve, (g) Chiasm, and (h) Eyes. Each panel shows scatter plots of manually contoured versus auto-contoured D_{max} values, with regression line, equation, and coefficient of determination (R^2). D_{max}, maximum dose
