# Supplementary material for: Cross‐anatomical evaluation of a deep‐learning auto‐contouring system: qualitative, geometric, and dosimetric validation
Source: J Appl Clin Med Phys. 2026 Jun 15;27(6):e70662. doi: 10.1002/acm2.70662 (PMC13269653; doi:10.1002/acm2.70662)
Supplement: Supplementary file 3 — Supporting Information: 2026‐09190‐sup‐0004‐SI_Figure‐S03.pdf [file ACM2-27-e70662-s005.pdf]

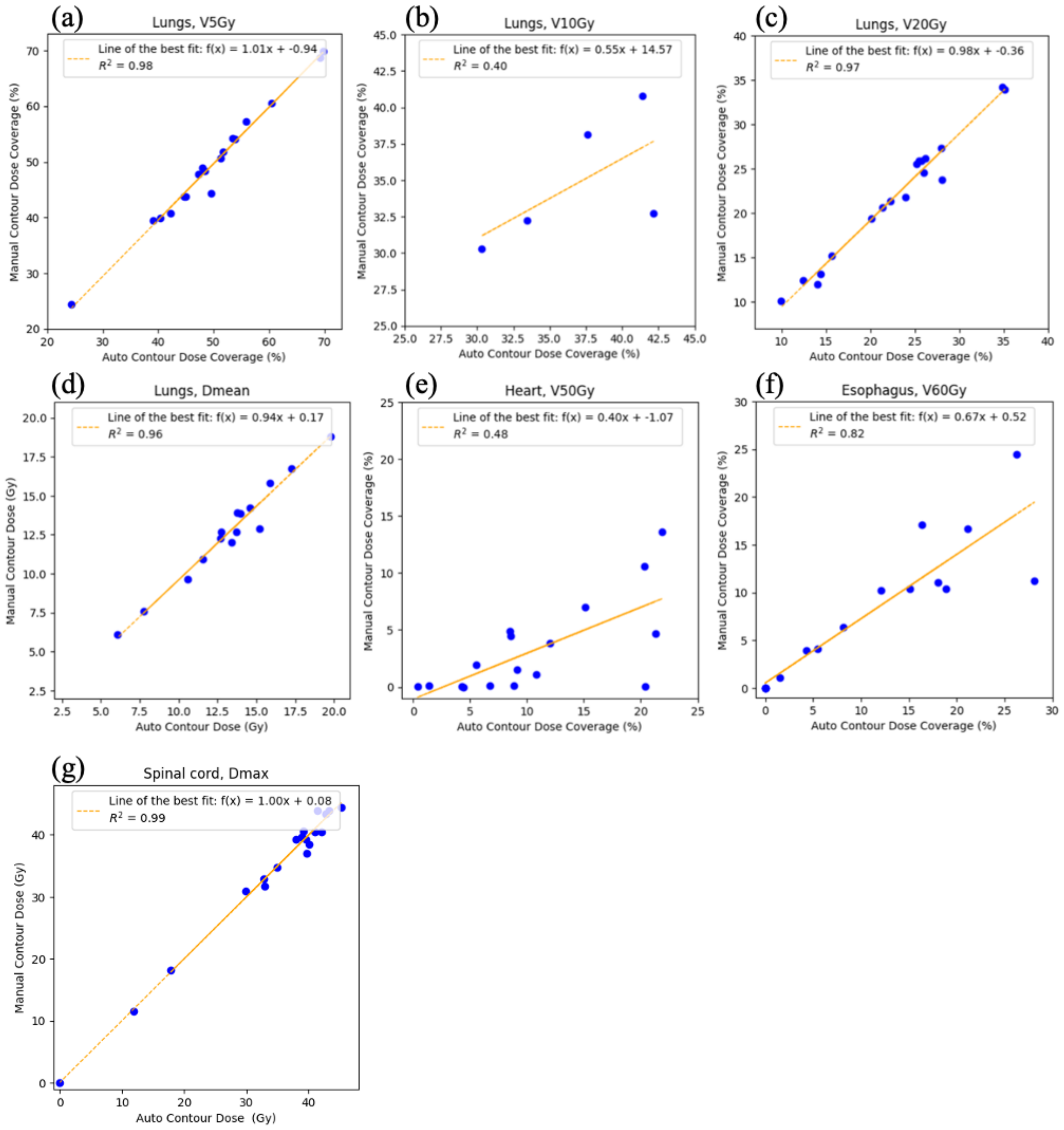

Supplementary Fig.3 Scatter plots comparing auto-contoured and manually contoured doses for organs at risk when planning radiotherapy in the thoracic region. (a) Lungs (V_{5Gy}), (b) Lungs (V_{10Gy}), (c) Lungs (V_{20Gy}), (d) Lungs (D_{mean}), (e) Heart (V_{50Gy}), (f) Esophagus (V_{60Gy}), and (g) Spinal cord (D_{max}). Each panel shows scatter plots of manually contoured versus auto-contoured dose metrics, with regression line, equation, and coefficient of determination (R^2). D_{max}, maximum dose; D_{mean}, mean dose
