# Supplementary material for: Cross‐anatomical evaluation of a deep‐learning auto‐contouring system: qualitative, geometric, and dosimetric validation
Source: J Appl Clin Med Phys. 2026 Jun 15;27(6):e70662. doi: 10.1002/acm2.70662 (PMC13269653; doi:10.1002/acm2.70662)
Supplement: Supplementary file 4 — Supporting Information: 2026‐09190‐sup‐0005‐SI_Figure‐S04.pdf [file ACM2-27-e70662-s007.pdf]

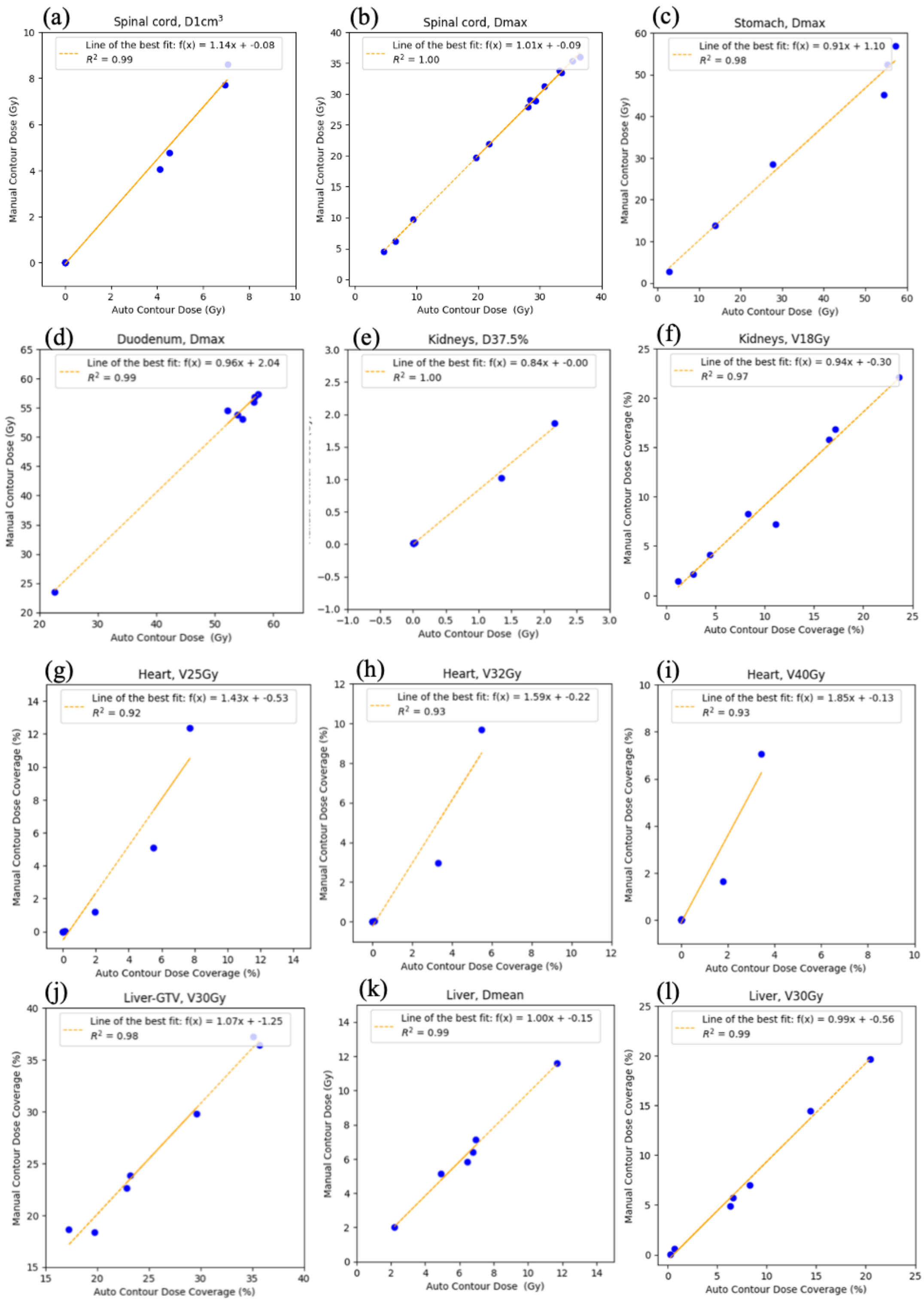

Supplementary Fig.4 Scatter plots comparing auto-contoured and manually contoured doses for organs at risk when planning radiotherapy in the abdominal region. (a) Spinal cord (D1cm³), (b) Spinal cord (D_{max}), (c) Stomach (D_{max}), (d) Duodenum (D_{max}), (e) Kidneys ($D_{37.5\%}$), (f) Kidneys (V_{18Gy}), (g) Heart

(V_{25Gy}), (h) Heart (V_{32Gy}), (i) Heart (V_{40Gy}), (j) Liver-GTV (V_{30Gy}), (k) Liver (D_{mean}), and (l) Liver (V_{30Gy}). Each panel shows scatter plots of manually contoured versus auto-contoured dose metrics, with regression line, equation, and coefficient of determination (R^2). D_{max}, maximum dose; D_{mean}, mean dose; GTV, gross tumor volume
