# Supplementary material for: Cross‐anatomical evaluation of a deep‐learning auto‐contouring system: qualitative, geometric, and dosimetric validation
Source: J Appl Clin Med Phys. 2026 Jun 15;27(6):e70662. doi: 10.1002/acm2.70662 (PMC13269653; doi:10.1002/acm2.70662)
Supplement: Supplementary file 5 — Supporting Information: 2026‐09190‐sup‐0006‐SI_Figure‐S05.pdf [file ACM2-27-e70662-s001.pdf]

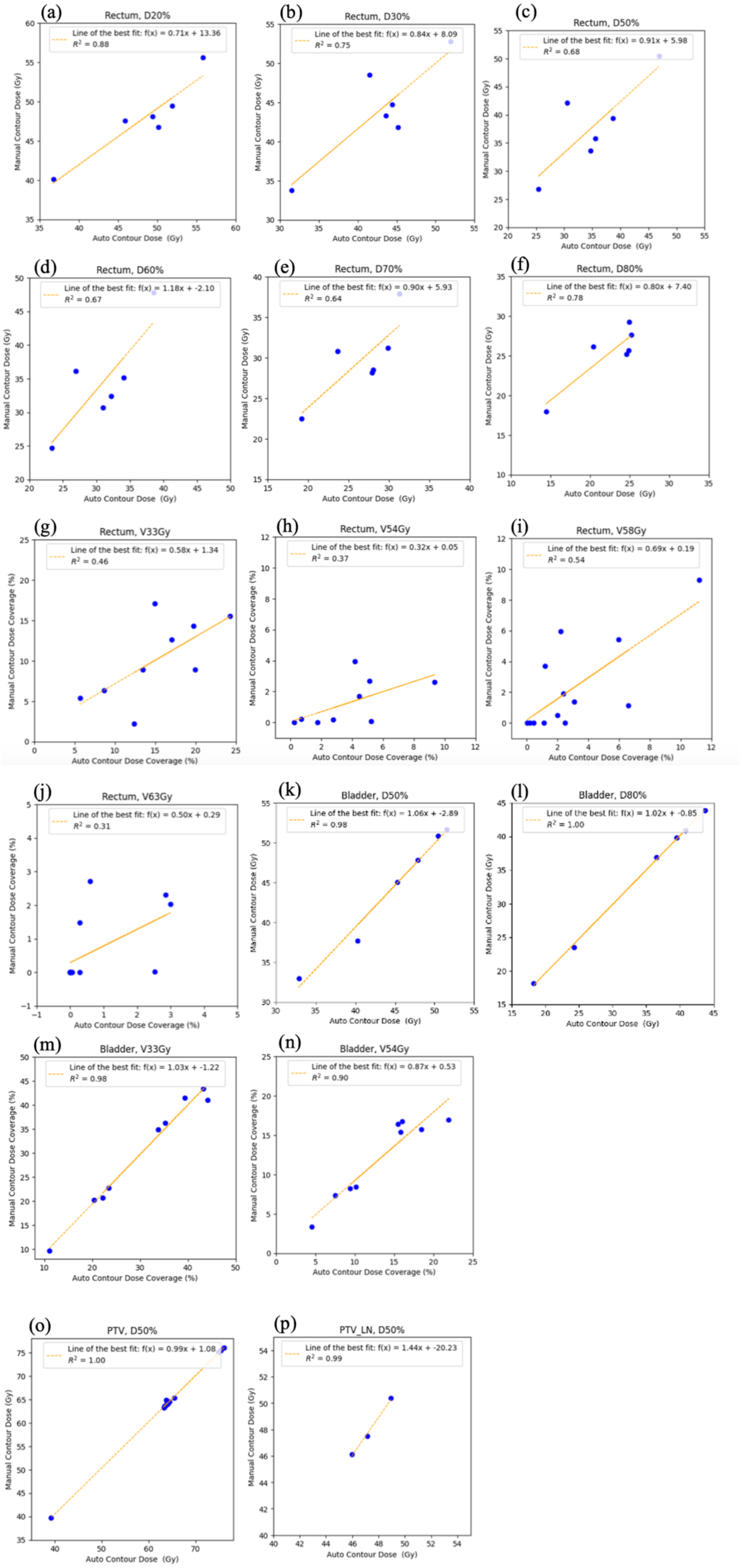

Supplementary Fig.5 Scatter plots comparing auto-contoured and manually contoured doses for organs at risk and PTVs when planning radiotherapy to the male pelvis. (a) Rectum ($D_{20\%}$), (b) Rectum ($D_{30\%}$), (c) Rectum ($D_{50\%}$), (d) Rectum ($D_{60\%}$), (e)

Rectum ($D_{70\%}$), (f) Rectum ($D_{80\%}$), (g) Rectum (V_{33Gy}), (h) Rectum (V_{54Gy}), (i) Rectum (V_{58Gy}), (j) Rectum (V_{63Gy}), (k) Bladder ($D_{50\%}$), (l) Bladder ($D_{80\%}$), (m) Bladder (V_{33Gy}), (n) Bladder (V_{54Gy}), (o) PTV ($D_{50\%}$), and (p) PTV_LN ($D_{50\%}$). Each panel shows scatter plots for manually contoured versus auto-contoured dose metrics, with regression line, equation, and coefficient of determination (R^2). The PTV was defined as the prostate clinical target volume expanded with the institutional margin, and PTV_LN was defined as the iliac lymph nodes (LN_Iliac) expanded with the institutional margin. LN, lymph nodes; PTV, planning target volume
