# Supplementary material for: Cross‐anatomical evaluation of a deep‐learning auto‐contouring system: qualitative, geometric, and dosimetric validation
Source: J Appl Clin Med Phys. 2026 Jun 15;27(6):e70662. doi: 10.1002/acm2.70662 (PMC13269653; doi:10.1002/acm2.70662)
Supplement: Supplementary file 6 — Supporting Information: 2026‐09190‐sup‐0007‐SI_Figure‐S06.pdf [file ACM2-27-e70662-s002.pdf]

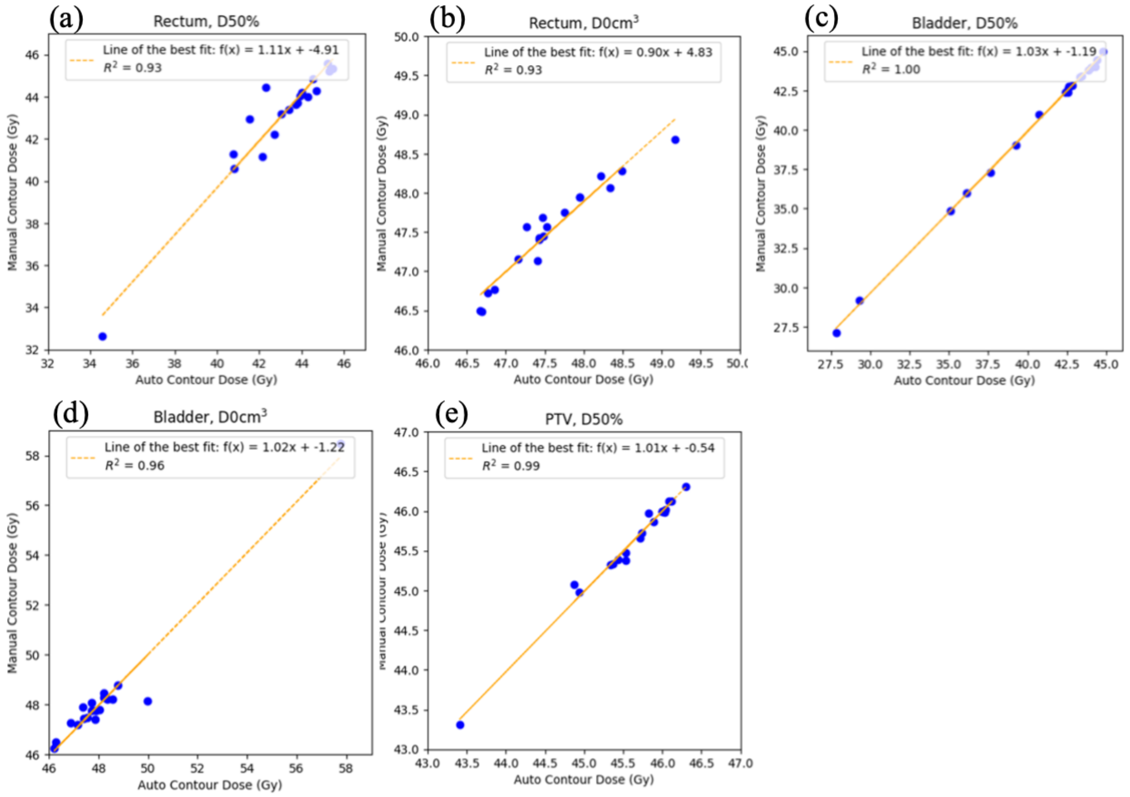

Supplementary Fig.6 Scatter plots comparing auto-contoured and manually contoured doses for organs at risk and PTVs when planning radiotherapy to the female pelvis. (a) Rectum (D50%), (b) Rectum (D 0cm^3), (c) Bladder (D50%), (d) Bladder (D0cm^3), and (e) PTV (D50%). Each panel shows scatter plots for manually contoured versus auto-contoured dose metrics, with regression line, equation, and coefficient of determination (R^2). PTV_LN was defined as the iliac lymph nodes (LN_Iliac) expanded with the institutional margin. LN, lymph nodes; PTV, planning target volume
