# Supplementary material for: Cross‐anatomical evaluation of a deep‐learning auto‐contouring system: qualitative, geometric, and dosimetric validation
Source: J Appl Clin Med Phys. 2026 Jun 15;27(6):e70662. doi: 10.1002/acm2.70662 (PMC13269653; doi:10.1002/acm2.70662)
Supplement: Supplementary file 7 — Supporting Information: 2026‐09190‐sup‐0008‐SI_Table‐S01.pdf [file ACM2-27-e70662-s004.pdf]

Supplementary Table 1. Dose constraints for organs at risk when planning stereotactic radiosurgery in the brain

**SRS** structure	metrics,criteria	Auto	Manual	Data points
Brainstem	D1cm^3<10Gy	18	18	19
Brainstem	D0%<15Gy	17	18	19
Spinal cord	D1.2cm^3<7Gy	9	9	9
Spinal cord	D0.3cm^3<10Gy	13	13	13
Spinal cord	D0%<14Gy	19	19	19
Optic pathway	D0.2cm^3<8Gy	16	16	16
Optic pathway	D0%<10Gy	17	17	17

SRS, stereotactic radiosurgery

Supplementary Table 2. Dose constraints for organs at risk when planning radiotherapy to the head and neck

**H&N**<br>structure	metrics,criteria	Auto	Manual	Data points
SpinalCord	Dmax≤45Gy	13	13	13
Brainstem	Dmax≤50Gy	13	13	15
Parotid_L	Dmean≤26Gy	6	6	11
Parotid_R	Dmean≤26Gy	7	7	10
Mandible	Dmax≤70Gy	6	6	10
Optic Nerve	Dmax≤70Gy	5	5	6
Chiasm	Dmax≤70Gy	7	7	7
Eyes	Dmax≤70Gy	7	7	8
Lens	Dmax≤10Gy	3	3	5

D_{max}, maximum dose; D_{mean}, mean dose; H&N, head and neck

Supplementary Table 3. Dose constraints for organs at risk when planning radiotherapy to the thoracic region

**Thorax**<br>structure	metrics,criteria	Auto	Manual	Data points
Lungs	V20Gy<35%	12	13	13
	V20Gy<30%	12	12	13
	V5Gy<70%	18	18	18
	V5Gy<60%	15	15	18
	Dmean<20Gy	15	15	15
	V20Gy<40%	5	5	5
	V20Gy<37%	5	5	5
	V10Gy<40%	3	4	5
	V15Gy<950cm^3	1	1	1
Lungs-GTV	V5Gy<60%	2	2	2
	V20Gy<35%	2	2	2
	V40Gy<100cm^3	1	1	1
	V15Gy<25%	1	1	1
	V20Gy<20%	1	1	1
	Dmean<18Gy	1	1	1
	V16Gy<37%	1	1	1
Heart	V50Gy<30%	17	17	17
	V50Gy<25%	17	17	17
	V45Gy<35%	3	3	3
	V45Gy<40%	1	1	1
	V36.6Gy<15cm^3	1	1	1
	Dmax<42.5Gy	1	1	1
Heart_03	V30Gy<15cm^3	1	1	1
Esophagus	V60Gy<19%	14	16	17
	V60Gy<17%	12	15	17
	D0cm^3<72Gy	2	2	2
	D0cm^3<110%	1	1	1
	V100%<17%	1	1	1
Spinal cord	Dmax<45Gy	16	17	17
	D0cm^3<48Gy	3	3	3
	D1cm^3<48Gy	1	1	1
	Dmax<36Gy	1	1	1
	V31Gy<5cm^3	1	1	1
	Dmax<38Gy	1	1	1
Spinal cord_03	Dmax<25Gy	1	1	1
	D2%<25Gy	1	1	1

D_{max}, maximum dose; D_{mean}, mean dose; GTV, gross tumor volume

Supplementary Table 4. Dose constraints for organs at risk when planning radiotherapy to the abdomen

**Abdomen** structure	metrics,criteria	Auto	Manual	Data points
Spinal cord	D1cm^3≤36Gy	6	6	6
	D1cm^3<20Gy	3	3	3
	Dmax<25Gy	3	3	3
	Dmax≤45Gy	6	6	6
	Dmax≤40.9Gy	4	4	4
Stomach	Dmin≤40Gy	4	4	4
	D5cm^3≤44Gy	4	4	4
	D1cm^3<20Gy	2	2	2
	Dmax<25Gy	2	2	2
	Dmax≤55Gy	2	3	4
	D30%<55Gy	4	4	4
Stomach05	Dmax≤58Gy	2	2	2
	V55Gy≤1%	2	2	2
Duo	D15cm^3≤40Gy	5	5	5
	D1cm^3<20Gy	1	1	1
	Dmax<25Gy	1	1	1
	Dmax≤58Gy	2	2	2
	V55Gy≤1%	2	2	2
	Dmax≤55Gy	1	1	4
	D30%<55Gy	4	4	4
Kidney	D37.5%≤14Gy	9	9	9
	V18Gy≤35%	4	4	4
	V18Gy≤65%	4	4	4
Kidney_L	V18Gy≤35%	1	1	1
Kidney_R	V18Gy≤35%	1	1	1
Heart	V25Gy≤20cm^3	6	5	6
	V32Gy≤10cm^3	6	6	6
	V40Gy≤2cm^3	6	6	6
	V48Gy<1cm^3	2	2	2
	V40Gy<10cm^3	2	2	2
Liver-GTV	V30Gy≤30%	5	5	7
	V20Gy<25%	3	3	3
	V15Gy<700cm^3	3	3	3
Liver	Dmean<15Gy	3	3	3
	Dmean≤30Gy	3	3	3
	V30Gy<40%	7	7	7
	V20Gy≤67%	4	4	4
Esophagus	D5cm^3≤40Gy	3	3	3
	D1cm^3<20Gy	3	3	3
	Dmax<25Gy	3	3	3

D_{max}, maximum dose; D_{mean}, mean dose; GTV, gross tumor volume

Supplementary Table 5. Dose constraints for organs at risk when planning radiotherapy to the male pelvis

**Male pelvic** structure	metrics,criteria	Auto	Manual	Data points
Rectum	V63Gy<3%	13	13	13
	V58Gy<20%	13	13	13
	V58Gy<10%	12	13	13
	V54Gy<25%	9	9	9
	V54Gy<17%	9	9	9
	V33Gy<35%	9	9	9
	D80%≤30Gy	6	6	6
	D70%≤40Gy	6	6	6
	D60%≤50Gy	6	6	6
	D50%≤60Gy	6	6	6
	D30%≤66Gy	6	6	6
	D20%≤70Gy	6	6	6
	V55Gy<25%	4	4	4
	V55Gy<17%	4	4	4
	V34Gy<35%	4	4	4
	V17Gy<50%	4	4	4
	V18.1Gy<50%	1	1	1
	V29Gy<20%	1	1	1
	V36Gy<1cm^3	1	1	1
Bladder	V54Gy<25%	9	9	9
	V33Gy<50%	9	9	9
	D80%≤50Gy	6	6	6
	D50%≤60Gy	6	6	6
	V55Gy<25%	4	4	4
	V34Gy<50%	4	4	4
	V18.1Gy<40%	1	1	1
	V37Gy<10cm^3	1	1	1
Femur_L	D0cm^3<45Gy	1	1	1
	D0%<50Gy	3	3	3
	D1cm^3<50%	3	3	3
Femur_R	D0cm^3<45Gy	1	1	1
	D0%<50Gy	3	3	3
	D1cm^3<50%	3	3	3
Femur	D0cm^3<45Gy	1	1	1
	D0%<50Gy	3	3	3
	D1cm^3<50%	3	3	3
PTV	D50%	—	—	20
PTV_LN	D50%	—	—	6

LN, lymph nodes; PTV, planning target volume

Supplementary Table 6. Dose constraints for organs at risk when planning radiotherapy to the female pelvis

**Female pelvic**<br>structure	metrics,criteria	Auto	Manual	Data points
Rectum	D50%<54Gy	19	19	19
	D50%<45Gy	16	16	16
	D0cm^3<55Gy	19	19	19
	D0cm^3<50Gy	19	19	19
Bladder	D50%<55Gy	19	19	19
	D50%<45Gy	19	19	19
	D0cm^3<57.5Gy	18	18	19
	D0cm^3<50Gy	18	18	19
PTV	D50%	—	—	19

PTV, planning target volume
